# Higher parental occupational social contact is associated with a reduced risk of incident pediatric type 1 diabetes: Mediation through molecular enteroviral indices

**DOI:** 10.1371/journal.pone.0193992

**Published:** 2018-04-17

**Authors:** Anne-Louise Ponsonby, Angela Pezic, Fergus J. Cameron, Christine Rodda, Andrew S. Kemp, John B. Carlin, Heikki Hyoty, Amirbabak Sioofy-Khojine, Terence Dwyer, Justine A. Ellis, Maria E. Craig

**Affiliations:** 1 Murdoch Children’s Research Institute, Royal Children’s Hospital, University of Melbourne, Flemington Rd, Parkville, Victoria, Australia; 2 National Centre for Epidemiology, Australian National University, Canberra, Australia; 3 Western Centre for Health Research and Education, Sunshine Hospital, St Albans, Victoria, Australia; 4 School of Medicine, Virology, University of Tampere, Lääkärinkatu, Finland; 5 The George Institute for Global Health, Oxford Martin School, University of Oxford, Oxford, United Kingdom; 6 Centre for Social and Early Emotional Development, Faculty of Health, Deakin University, Burwood, Victoria, Australia; 7 School of Women’s and Children’s Health, University of New South Wales, New South Wales, Australia; 8 Discipline of Child and Adolescent Health, University of Sydney, New South Wales, Australia; University of British Columbia, CANADA

## Abstract

We aimed to examine the association between parental occupational social contact and hygiene factors on type 1 diabetes (T1D) risk and possible mediation of these effects through child enteroviral infection. We interviewed 333 incident T1D cases and 660 controls from 2008–2011 in Melbourne, Australia. Enteroviral indices (ribonucleic acid by reverse transcription polymerase chain reaction and Coxsackie B virus antibody levels) in peripheral blood were measured in nested case control samples. Parent occupational social contact was assessed by the number of well or sick children, adults or animals contacted daily through work. Higher parental occupational social contact was strongly associated with reduced T1D risk with evidence of dose response (contact with the well or sick score, Adjusted odds ratio (AOR) per category: 0.73 (95% Confidence Interval (CI): 0.66, 0.81); P<0.001 or AOR 0.63 (95% CI: 0.53, 0.75); P<0.001) respectively). Nine of the ten parental social contact indices, were significant mediated through one or more enteroviral indices. The strength of association between enterovirus presence and T1D onset increased with child age (1.2 fold increase per year; P = 0.05). Lower child hand hygiene enhanced the adverse effect of low parental occupational contact with the sick; Synergy Index 5.16 (95% CI: 3.61, 7.36). The interaction between hand washing and parental occupational contact is more consistent with protection against parental enteroviral shedding than the sharing of a protective infectious agent or microbiome.

## Introduction

The incidence of paediatric type 1 diabetes mellitus (T1D) has increased over time [1]. This autoimmune disease has a first stage of preclinical autoimmunity and a second stage of clinical onset [2]. Meta-analysis indicates the presence of enterovirus (EV) by polymerase chain reaction (PCR) in peripheral blood is associated with a summary odds ratio of 9.8 (95% Confidence Interval (CI): 5.5, 17.4) for clinical TID onset [3]. EV is also markedly more commonly detected among the peripheral blood of family members (63% of parents; 60% of siblings) of incident T1D cases compared to only 3% and 0% of non-family child and adult controls respectively [4]. EV genome can be eliminated relatively quickly from peripheral blood [5]. In contrast, EV genome may be present in host gut mucosa and pancreatic islets for many years, leading to persistent disease with viral shedding [6] [7]. Prolonged EV elimination in faeces has been postulated to be responsible for T1D clustering among sibsets [4].

The role of EV infection in T1D is complex. T1D incidence has particularly increased in modern populations where EV is less prevalent [8]. Two possible mechanisms include:- (i) that in such populations EV is acquired at a later age which leads to adverse consequences and/or (ii) that the infectious contact load is reduced in such populations, leading to reduced ‘herd immunity’ (partly due to lack of maternal enterovirus antibodies in new-borns) and adverse immune consequences upon EV exposure. EV infection during the first year of life has been associated with a reduced risk of T1D onset [9]. However, to date, no study has demonstrated that the adverse effect of EV on T1D onset significantly increases with increasing age. The second mechanism has been difficult to investigate for T1D but occupational social contact (daily contact with a number of children, adults or animals through work) has been used as a proxy for investigating herd immunity issues for other diseases [10] [11].

High social contact occupations are associated with a greater infection rates [12] and re-boosting of established immune responses against pathogens [13]. Such re-exposure is particularly valuable for short term host immune responses and/or persistent infections [14]. EV may meet this criteria [6, 7] and Varicella-zoster virus does:- higher adult occupational social mixing or contact with children is associated with a reduced risk of herpes zoster in adulthood, likely mediated through boosted humoral immunity against latent Varicella-zoster virus [15]. High paternal occupational social contact is associated with maternal primary cytomegalovirus infection during pregnancy [16]. Apart from one small study [17], parental social contact has never been systematically evaluated for T1D.

The purpose of this report was to evaluate (i) whether higher parental occupational social contact with well and sick adults, children and animals was associated with a reduced risk of T1D, (ii) whether any such effect was modified by child hand hygiene before meals, and (iii) the extent that any apparent beneficial effect of higher parental occupational social contact on child T1D onset is mediated through altered EV indices at T1D onset. We also consider these findings in the context of age of T1D onset.

## Methods

### Cases

Participants with incident T1D were recruited between March 2008 and March 2011 at the Royal Children’s Hospital and Monash Medical Centre, Melbourne, Australia [18]. Inclusion criteria were participants with newly diagnosed T1D aged 1 to 14 years inclusive (Table 1).

**Table 1:** Characteristics of children in the early environment and type 1 diabetes prevention project.

Factor	Cases Mean (SD) or % (n/N)	Controls Mean (SD) or % (n/N)	P-value
**Age at recruitment (years)**	8.4 (3.6)	6.5 (3.5)	<0.001
**Maternal age at child’s birth (years)**	30.5 (4.8)	29.4 (5.5)	0.001
**Child ever breastfed (yes)**	85.9 (279/325)	80.4 (510/634)	0.038
**Male sex**	50.8 (169/333)	59.6 (393/660)	0.008
**Family history of insulin dependent diabetes mellitus**	15.8 (50/316)	12.8 (77/600)	0.213
**Caucasian**	91.5 (300/328)	78.8 (402/510)	<0.001

### Controls

Controls were recruited between January 2008 and July 2012 from the Royal Children’s Hospital day surgery unit which they attended for a minor surgical procedure. The healthy control children were aged 14 years or under and born in the state of Victoria, Australia. A range of minor reasons for surgery were targeted for inclusion [19]. These controls were recruited as part of the larger paediatric autoimmune disease platform.

Cases and controls with a major congenital abnormality or an illness that would forgo usual school attendance in the year prior to recruitment were excluded from study interview which involved parental questionnaire and clinical examination. A comprehensive questionnaire which included infection, demographic, lifestyle and environmental history over the child’s life course was obtained at a single interview. This included ancestry by grandparents’ racial origin, child sun exposure [20] and current child hygiene practices [21]Current weekday parental occupational social exposure to children, adults or animals and whether these groups were sick or well was recorded (S1 Table), following the approach of Thomas et al [10]. This approach was chosen as it provides quantitative responses, allowing dose response trends to be better evaluated.

Birth dates of all participating children and their siblings were used to provide sibling number and inter-sibling interval. Composite scores for contact with sick people or well people and animals were constructed as outlined in Table 2. Ethical approval was obtained from the Royal Children’s Hospital and the Monash Medical Centre Human Research Ethics Committees. Written consent was obtained from parents and assent from children aged 12 years and over.

**Table 2:** Higher parent occupational microbial contact is associated with a reduced risk of type 1 diabetes onset: Ten measures and two composite indices.

Category	Cases, % (n/N)	Controls, % (n/N)	AOR*	95% CI*	P-value	AOR†	95% CI†	P-value
**Contact with well adults, mother**								
Not at all	14.7% (45/307)	4.8% (26/540)	Ref			Ref		
n = < 10	34.5% (106/307)	40.7% (220/540)	0.25	0.15, 0.44	<0.001	0.11	0.05, 0.25	<0.001
n = 10 < 30	29.6% (91/307)	28.9% (156/540)	0.27	0.15, 0.49	<0.001	0.10	0.04, 0.23	<0.001
n = 30 or more	21.2% (65/307)	25.6% (138/540)	0.20	0.11, 0.37	<0.001	0.09	0.04, 0.22	<0.001
			*Test of trend*		*<0*.*001*			*<0*.*001*
**Contact with well adults, father**								
Not at all	3.3% (10/307)	1.5% (7/477)	Ref			Ref		
n = < 10	21.5% (66/307)	23.7% (113/477)	0.35	0.12, 1.01	0.05	0.17	0.04, 0.73	0.02
n = 10 < 30	45.9% (141/307)	35.4% (169/477)	0.54	0.19, 1.52	0.24	0.26	0.06, 1.11	0.07
n = 30 or more	29.3% (90/307)	39.4% (188/477)	0.29	0.10, 0.83	0.02	0.15	0.04, 0.65	0.01
			*Test of trend*		*0*.*06*			*0*.*01*
**Contact with well children, mother**								
Not at all	30.4% (93/306)	10.9% (57/521)	Ref			Ref		
n = < 10	42.2% (129/306)	55.1% (287/521)	0.24	0.16, 0.36	<0.001	0.14	0.08, 0.25	<0.001
n = 10 < 30	18.3% (56/306)	19.4% (101/521)	0.31	0.19, 0.50	<0.001	0.17	0.09, 0.31	<0.001
n = 30 or more	9.2% (28/306)	14.6% (76/521)	0.16	0.09, 0.28	<0.001	0.09	0.05, 0.19	<0.001
			*Test of trend*		*<0*.*001*			*<0*.*001*
**Contact with well children, father**								
Not at all	39.7% (121/305)	26.6% (119/448)	Ref			Ref		
n = < 10	50.2% (153/305)	60.5% (271/448)	0.52	0.37, 0.72	<0.001	0.43	0.29, 0.63	<0.001
n = 10 < 30	7.5% (23/305)	6.7% (30/448)	0.74	0.40, 1.38	0.35	0.70	0.33, 1.49	0.36
n = 30 or more	2.6% (8/305)	6.3% (28/448)	0.25	0.11, 0.58	0.001	0.21	0.08, 0.54	0.001
			*Test of trend*		*<0*.*001*			*<0*.*001*
**Contact with well animals, mother**								
Not at all	56.7% (174/307)	39.7% (204/514)	Ref			Ref		
n = < 10	40.1% (123/307)	58.6% (301/514)	0.44	0.33, 0.60	<0.001	0.33	0.23, 0.48	<0.001
n = 10 or more	3.3% (10/307)	1.8% (9/514)	1.12	0.43, 2.88	0.82	0.60	0.23, 1.57	0.30
			*Test of trend*		*<0*.*001*			*<0*.*001*
**Contact with well animals, father**								
Not at all	49.0% (150/306)	44.1% (198/449)	Ref			Ref		
n = < 10	48.0% (147/306)	52.8% (237/449)	0.73	0.54, 0.99	0.05	0.60	0.42, 0.86	0.006
n = 10 or more	2.9% (9/306)	3.1% (14/449)	0.92	0.38, 2.24	0.85	0.84	0.31, 2.23	0.72
			*Test of trend*		*0*.*09*			*0*.*02*
**Any contact with sick adults, mother**								
No	83.0% (253/305)	75.6% (380/503)	Ref			Ref		
Yes	17.1% (52/305)	24.5% (123/503)	0.58	0.40, 0.84	0.004	0.51	0.34, 0.78	0.002
**Any contact with sick adults, father**								
No	89.6% (268/299)	79.6% (356/447)	Ref			Ref		
Yes	10.4% (31/299)	20.4% (91/447)	0.43	0.27, 0.68	<0.001	0.33	0.20, 0.55	<0.001
**Any contact with sick children, mother**								
No	88.0% (270/307)	76.8% (381/496)	Ref			Ref		
Yes	12.1% (37/307)	23.2% (115/496)	0.43	0.28, 0.65	<0.001	0.36	0.23, 0.56	<0.001
**Any contact with sick children, father**								
No	95.9% (282/294)	87.6% (381/435)	Ref			Ref		
Yes	4.1% (12/294)	12.4% (54/435)	0.25	0.13, 0.49	<0.001	0.15	0.07, 0.33	<0.001
**Composite any contact with well adults, children or animals, mother and father (occupational well score)** ‡								
Not at all	2.5% (8/325)	1.4% (8/562)	Ref			Ref		
Category 1	8.0% (26/325)	4.5% (25/562)	1.03	0.33, 3.29	0.95	2.92	0.51, 16.86	0.23
Category 2	12.9% (42/325)	13.9% (78/562)	0.53	0.18, 1.56	0.25	0.59	0.14, 2.53	0.48
Category 3	24.9% (81/325)	15.8% (89/562)	0.72	0.25, 2.08	0.55	0.40	0.10, 1.63	0.20
Category 4	14.2% (46/325)	21.2% (119/562)	0.38	0.13, 1.09	0.07	0.20	0.05, 0.83	0.03
Category 5	7.4% (24/325)	7.8% (44/562)	0.46	0.15, 1.42	0.18	0.17	0.04, 0.75	0.02
Category 6	30.2% (98/325)	35.4% (199/562)	0.43	0.15, 1.22	0.11	0.20	0.05, 0.78	0.02
			*Test of trend*		*0*.*002*			*<0*.*001*
**Composite any contact with sick adults or children, mother and father (occupational sick score)** §								
Not at all	76.5% (244/319)	62.8% (329/524)	Ref			Ref		
Category 1	11.6% (37/319)	15.7% (82/524)	0.55	0.35, 0.85	0.007	0.53	0.32, 0.87	0.01
Category 2	7.5% (24/319)	12.8% (67/524)	0.46	0.28, 0.77	0.003	0.38	0.22, 0.68	0.001
Category 3	2.8% (9/319)	3.2% (17/524)	0.69	0.30, 1.62	0.40	0.45	0.19, 1.09	0.08
Category 4	1.6% (5/319)	5.5% (29/524)	0.20	0.08, 0.54	0.001	0.11	0.03, 0.36	<0.001
			*Test of trend*		*<0*.*001*			*<0*.*001*

Ref **=** Reference category. n = number of person-specific or animal-specific contacts.

* Adjusted for age and sex only

† Adjusted for age, sex, family history of insulin dependent diabetes mellitus, time spent in sun during last winter weekdays, ever breastfed, maternal age at birth, SEIFA disadvantage index and Caucasian ancestry

‡ Occupational well score is a summation of individual occupational well categories. 0 = no exposure in all the six categories, 2 = some exposure in 2 of the 6 categories etc.

§ Occupational sick score is a summation of individual occupational sick categories. 0 = no exposure in all the four categories, 2 = some exposure in 2 of the 4 categories etc.

### Blood samples

For T1D cases, serum samples were obtained at time of admission and blood samples were obtained again at interview (median sampling time after initial admission to blood draw, 0.4 (IQR = 0.1 to 6.6) weeks). Control children provided a venous blood sample collected at insertion of the peripheral line for day surgery. Case and control blood samples were separated into heparinized plasma and peripheral blood mononuclear cells. Plasma was stored in 1 ml aliquots in a -80°C facility.

### Enteroviral indices

To allow direct matching by sex and within a year of age, nested case control samples were randomly selected for viral studies. Case admission serum and control plasma samples were tested for detectable EV ribonucleic acid by one step quantitative real time reverse transcription polymerase chain reaction with SYBR green dye using the LightCycler RNA Amplification Kit SYBR Green I (Product No. 12015137001, Roche applied systems, USA) [22] on the LightCycler 2.0 Instrument (Roche Diagnostics, USA) at the South Eastern Area Laboratory Services at Prince of Wales Hospital, as previously described [22]. Multiplex real time-polymerase chain reaction (PCR) for EV, herpes simplex 1, Epstein-Barr virus, Varicella-zoster virus and cytomegalovirus detection was also conducted [23]. Neutralizing antibodies were measured against Coxsackie B1 virus (CVB) (American Type Culture Collection prototype strain) with a plaque neutralization assay at the Department of Virology, University of Tampere, Finland [24]. The plasma sample was first mixed with 100 plaque-forming units of the virus and incubated for 1 h at 37°C followed by overnight incubation at room temperature. This mixture was then transferred to a monolayer of green monkey kidney cells on six-well plastic plates (Nunclon, ThermoFisher Scientific, product No. 140685) in plaque assay medium containing minimal essential medium supplemented with 1% FBS, 40 U/mL penicillin-streptomycin, 0.0023% glucose, 1 X L-glutamine, 1.5 mmol/L MgCl2, and 1.5 mmol/L carboxymethyl cellulose (HEPES). The number of virus-generated plaques was counted manually after 48 h of incubation at 37°C. All test runs included both virus-positive and virus-negative control wells. The final dilutions of plasma in the assay were 1/4 and 1/16, and the sample was judged seropositive if either of these dilutions inhibited at least 75% of the plaques. The range of inhibition was 0 to 100%. Detection of neutralizing antibodies in plasma in such titers has been shown to be a reliable marker of past infection [25]. We examined the case serum samples at first presentation because timing of EV infection in relation to T1D disease course is very important [26]. Although serum and plasma samples provide comparable Immunoglobulin G (IgG) measures be enzyme-linked immunosorbent assay [27, 28] and very similar for viral PCR measures [29], some studies have had lower viral detection levels in serum than plasma [30, 31]. Thus, the excess proportion of cases with detectable EV to controls could possibly be even a little higher than reported.

### Statistical methods

Characteristics of the cases and controls are presented as mean (standard deviation) or percentages. Sibling birthdates were used to obtain the number of age-specific siblings. Sibling-years, defined as the total number of years a child had been exposed to any siblings, regardless of sibling age (up to age 18), was calculated for the subject at time of interview [19] and reconstructed for past ages of the subjects, for example, at age 2 years.

Multivariable logistic regression was used to examine case-control differences. Adjusted odds ratios (AOR) and 95% CI are reported. All AORs were adjusted for age at recruitment and sex, and then additionally for other factors such as family history of T1D, Caucasian ancestry, ever breastfed, maternal age at birth, skin type, low sun exposure in past winter on weekends and the socioeconomic indexes for areas disadvantage index [32]. These factors were included as covariates as they potentially confounded the association between parental occupational social contact and T1D onset. Tests for trend with categorical covariates were undertaken by using a single predictor taking category rank scores, based on the Wald test.

To assess interaction on the multiplicative scale, we added product terms to the logistic models. To assess interaction on the additive scale, we focussed on the Synergy Index as this allows confounding to be considered also [33]. Mediation analysis was undertaken to determine if EV infection was a likely intermediate factor in a causal pathway between the selected proxy microbial exposures (parental occupational social contact, child attending day care etc.) and T1D onset [34]. We followed the methods of VanderWeele [35] and assessed two EV indices:- EV presence by PCR and antibodies against EV serotypes previously linked with T1D as EV infection biomarkers.

Multiple linear regression was used to assess the influence of environmental factors on age of onset in completed years, after first accounting for constitutional factors of ancestry, sex and parental history of T1D. The interaction between age of onset, EV presence and T1D risk was assessed by adding a product term and assessing the reduction in deviance using the log likelihood ratio test. Alternative models allowing age to be categorized in a non-linear form were also developed and compared using the log likelihood ratio test. The common odds ratio test was also used to assess whether the association between the EV indices and T1D onset varied by age in the age and sex matched analyses [36]. We conducted an additional analysis aimed to recalculate the main study findings using an estimation method to better reflect all Victorian births. Cases born outside Victoria were excluded. For controls, inverse probability weighting was used to re-weight the available controls to better reflect the entire Victorian paediatric population. Weights were calculated as the inverse of the probability of the controls being selected for the study [37] compared to 99.9% of live births in the same birth year, available from the Victorian Perinatal Data Collection Unit. Probability of selection was modelled using month of birth, birth weight, gestational age at birth, maternal marital status, mode of delivery, maternal age and SEIFA disadvantage index. We used Stata 14.1 software (StataCorp, College Station, TX) for all analyses [38].

## Results

333 cases (83% of incident cases) and 660 controls (a participation rate of 82%) were involved. Table 1 shows that the cases had a mean age of 8.4 (SD 3.6) years and 50.8% were male. The control mean age was 6.5 (SD 3.5) years and 59.6% were male. In our setting, day care was associated with high child contact, with 86% (102/118) of control children at day care being exposed to 11 or more children in the same room.

### The inverse association between parental occupational social contact and T1D onset

Higher parental occupational social contact was strongly associated with reduced T1D risk (Table 2).

The magnitude of these inverse associations were high, with evidence of dose response. The inverse associations were consistently evident across all ten exposure categories: Higher parental occupational social contact was strongly associated with reduced T1D risk with evidence of dose response (AOR per category of increasing contact with the well or sick score AOR (0.73 (95% CI: 0.66, 0.81); *P*<0.001 or AOR 0.63 (95% CI: 0.53, 0.75); *P*<0.001) respectively. The magnitude of effect was substantial. For example, the highest category of composite well or sick parental occupational score was associated with a more than five-fold or ten-fold reduction in T1D risk, respectively, compared to no exposure.

### The association between child hand hygiene, day care and other factors and T1D onset

**Fig 1:**
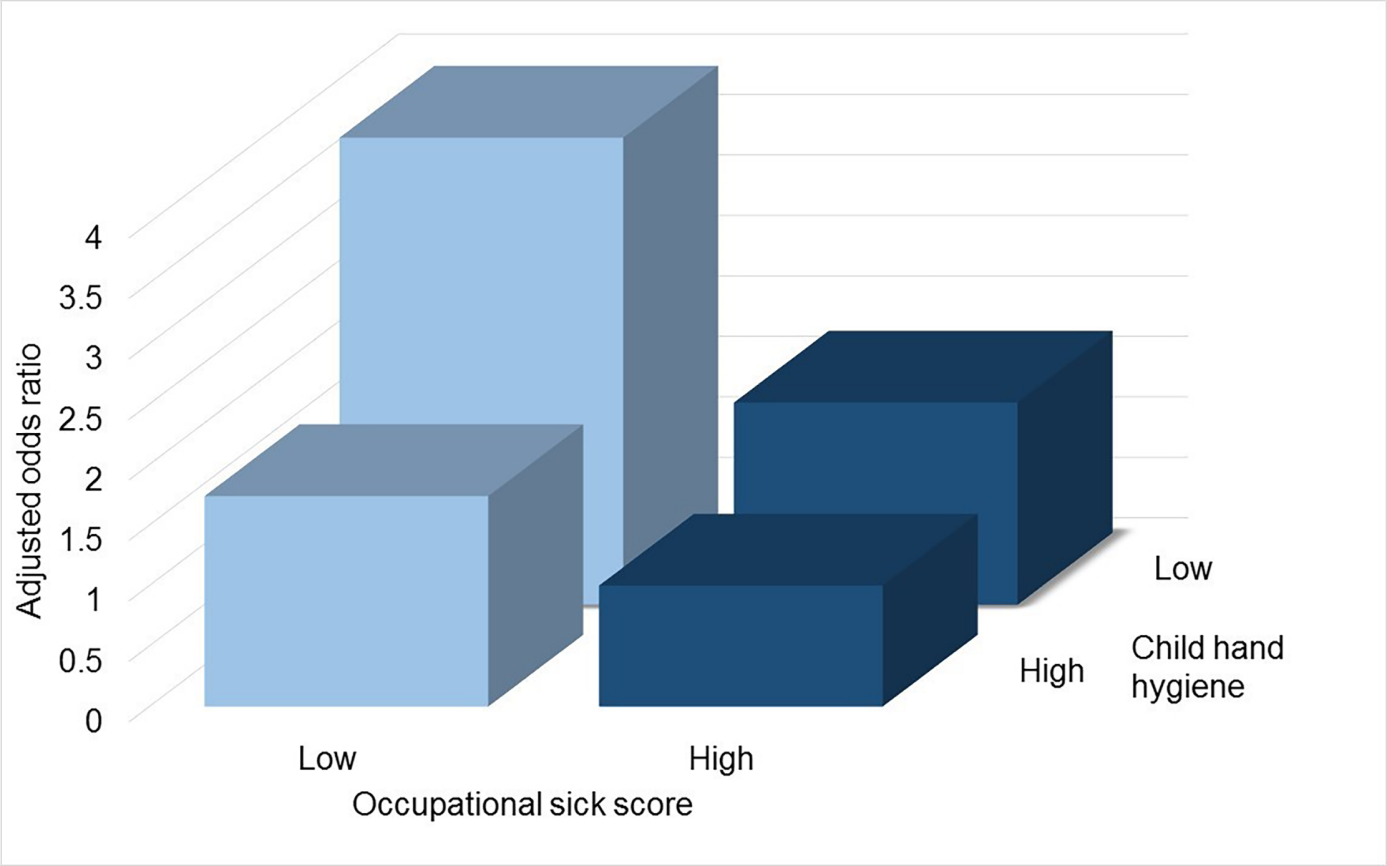
Combined exposure to low occupational sick score and low hand washing is associated with greater risk of type 1 diabetes onset: Evidence of interaction. The combined exposure to low occupational sick score (0–2 vs. rest) and low hand washing (never, occasionally) had an odds ratio of 3.86 (95% CI: 2.08, 7.16); among those with a low occupational sick score and high hand washing the odds ratio was 1.74 (95% CI: 0.89, 3.41); among those with low hand washing and a high occupational sick score the odds ratio was 1.67 (95% CI: 0.86, 3.24) compared to the lowest risk category associated with both high occupational sick score and high hand washing (AOR 1.00 (reference)). The Synergy Index is 5.16 (95% CI: 3.61, 7.36) with a Relative Excess Risk due to Interaction of 37.46 (95% CI: 13.96, 60.95) and Attributable Proportion of 0.79 (95% CI: 0.72, 0.86). The AOR_multi_ is 1.19, P = 0.03. All odds ratio adjusted for age and sex. Thus, the interaction is evident on the additive and multiplicative scale.

Better hand hygiene before meals was also strongly associated with reduced T1D risk, with evidence of dose response (Table 3). Day care was also associated with reduced T1D risk. (Table 3). Of interest, better child hand hygiene, as reported in Fig 1, was associated with the child having less colds or flu (AOR 0.64, P = 0.003),but not significantly less gastroenteritis (AOR 0.83, P = 0.16) over the past year.

**Table 3:** The association between child hygiene, day care and recent illness and type 1 diabetes onset.

	Cases, % (n/N)	Controls, % (n/N)	AOR*	95% CI*	P-value	AOR†	95% CI†	P-value
**Hand washing before meals frequency **								
**Never**	3.0% (10/330)	1.3% (8/635)	Ref			Ref		
**Occasionally**	17.6% (58/330)	10.2% (65/635)	0.74	0.26, 2.09	0.57	0.83	0.22, 3.09	0.78
**Sometimes**	23.0% (76/330)	16.5% (105/635)	0.59	0.21, 1.63	0.31	0.62	0.17, 2.26	0.47
**Usually**	35.5% (117/330)	36.7% (233/635)	0.46	0.17, 1.24	0.13	0.56	0.16, 1.98	0.37
**Always**	20.9% (69/330)	35.3% (224/635)	0.28	0.10, 0.76	0.01	0.37	0.10, 1.32	0.12
** **			* Test of trend*		*<0*.*001*			*0*.*002*
**Child attended day care in the past year**								
**No**	87.8 (280/319)	79.7 (463/581)	Ref			Ref		
**Yes**	12.2 (39/319)	20.3 (118/581)	0.64	0.43, 0.96	0.03	0.52	0.33, 0.81	0.004
**Flu or cold in the past 12 months**								
**No**	27.2 (88/323)	48.9 (278/569)	Ref			Ref		
**Yes**	72.8 (235/323)	51.1 (291/569)	2.70	1.98, 3.68	<0.001	2.58	1.80, 3.71	<0.001

Ref = Reference category

* Adjusted for age and sex only

† Adjusted for age, sex, family history of insulin dependent diabetes mellitus, time spent in sun during last winter weekdays, ever breastfed, maternal age at birth, SEIFA disadvantage index and Caucasian ancestry

### Poorer child hygiene before meals exacerbates the association between low parent occupational contact and a higher risk of type 1 diabetes onset

The association between parental occupational social contact and T1D differed by child hand hygiene standards. Fig 1 shows the lowest risk was for those with high parental occupational social contact and also high hand hygiene and that the combined risk of both low parental occupational social contact and also low hand hygiene before meals was greater than expected. For parental occupational well score (0–1) and poor child hand hygiene before meals (never, occasionally), the Synergy Index was 1.22 (95% CI: 0.39, 3.87) and parental occupational sick score (none) and poor child hand hygiene before meals (never, occasionally), the Synergy Index was 5.16 (95% CI: 3.61, 7.36) (Fig 1).

#### Enterovirus indices

EV was detected by PCR more commonly among T1D cases than controls, with an adjusted odds ratio of 5.61 (95% CI: 3.16, 9.98). T1D cases had higher EV IgG levels (Table 4). The correlation between detectable EV and EV seropositivity was r = -0.01; P *=* 0. 89 and r = 0.03, P *=* 0.57 for cases and controls respectively.

**Table 4:** The association between enteroviral indices and type 1 diabetes onset in childhood.

	Cases, % (n/N) or proportion (95% CI)%	Controls, % (n/N) or proportion (95% CI)%	AOR*	95% CI*	P-value	AOR†	95% CI†	P-value
**Prevalence of neutralizing antibodies in 1:4 serum dilution**								
	0.88 (0.85, 0.92)%	0.81 (0.78, 0.85)%	n/a	n/a	0.02	n/a	n/a	0.047
**Prevalence of neutralizing antibodies in 1:16 serum dilution**								
	0.59 (0.52, 0.66)%	0.54 (0.49, 0.59)%	n/a	n/a	0.50		n/a	0.88
**EV antibodies present**								
**No**	12.8% (20/156)	21.0% (68/324)	Ref			Ref		
**Yes**	87.2% (136/156)	79.0% (256/324)	1.65	0.95, 2.87	0.07	1.49	0.84, 2.65	0.18
**Enterovirus detectable by LightCycler PCR**								
**No**	78.0% (230/295)	94.7% (484/511)	Ref			Ref		
**Yes**	22.0% (65/295)	5.3% (27/511)	5.07	3.09, 8.31	<0.001	5.61	3.16, 9.98	<0.001

Ref = Reference category

* Adjusted for age and sex

† Adjusted for age, sex, Caucasian ancestry and family history of insulin dependent diabetes mellitus.

Among controls, day care attendance associated with a five-fold increase in the likelihood of EV presence by PCR (AOR 5.30 (95% CI: 1.27, 22.10); P *=* 0.02). Further, day care attendance was associated with EV seropositivity (AOR 2.72 (95% CI: 1.07, 6.95); P *=* 0.04).

### Mediation analyses—the association between higher parental occupational well or sick contact on type 1 diabetes is mediated through altered enteroviral indices in the child

Restricting to the nested viral study, parental occupational well or sick contact was again strongly associated with reduced T1D risk. For mediation, highly consistent patterns were seen across the ten exposure categories (Table 5).

**Table 5:** The percentage of selected factors associated with type 1 diabetes that are mediated through enteroviral indices.

		Enterovirus detected			Enterovirus seropositivity	
Factor	N	% of total effect mediated	95% CI	N	% of total effect mediated	95% CI
Occupational contact with well adults, mother	**698**	**3.8**	**2.9, 6.4**	**423**	**2.1**	**1.6, 3.7**
Occupational contact with well adults, father	646	-13.4	-211.4, 224.4	377	9.9	-100.8, 154.3
Occupational contact with well children, mother	**681**	**0.1**	**0.1, 0.2**	**415**	**1.2**	**1.0, 1.7**
Occupational contact with well children, father	**622**	**0.9**	**0.6, 2.0**	**368**	**2.7**	**2.0, 4.4**
Occupational contact with well animals, mother	**679**	**4.8**	**3.5, 7.7**	**418**	**1.1**	**0.1, 1.5**
Occupational contact with well animals, father	624	-7.3	-31.1, 35.0	**372**	**1.9**	**1.2, 5.3**
Occupational contact with sick adults, mother	**664**	**-0.4**	**-1.0, -0.3**	**402**	**10.2**	**5.7, 42.4**
Occupational contact with sick adults, father	**611**	**6.1**	**4.4, 11.5**	**360**	**3.1**	**2.3, 5.6**
Occupational contact with sick children, mother	**659**	**1.4**	**1.0, 2.5**	**396**	**3.3**	**2.2, 7.8**
Occupational contact with sick children, father	**596**	**3.2**	**2.4, 5.8**	**350**	**-0.6**	**-1.3, -0.4**

Five of the six indicators of parental occupational well contact were demonstrated to be significantly mediated through EV PCR presence and/or also EV seropositivity. The mediated fractions were not large in magnitude but the 95% confidence intervals excluded a zero mediation value. Similarly, all four indicators of parental occupational sick contact were demonstrated to be significantly mediated through either EV PCR presence and/or EV seropositivity. A greater portion of mediated effect was accounted for by variation in EV presence than EV seropositivity. However, there was an anomalous finding where inverse association between maternal contact sick adults was not partly accounted for by mediation in EV presence, rather the reverse. However, this factor did appear to be partly mediated through EV seropositivity. The associations between child hand hygiene or day care and T1D onset were not demonstrated to be directly mediated through variation in EV presence or EV seropositivity. There was no interaction between parental occupational contact, EV infection and T1D.

### Age of onset of type 1 diabetes

Day care attendance was associated with a younger age of T1D onset (mean difference, 2.49 (95% CI: 1.29, 3.69) years) and also associated with younger age among controls (mean difference, 1.15 (95% CI: 0.37, 1.94 years). Increasing composite score for parental occupational well or sick contact and child hand hygiene were not associated with age of T1D onset.

**Fig 2:**
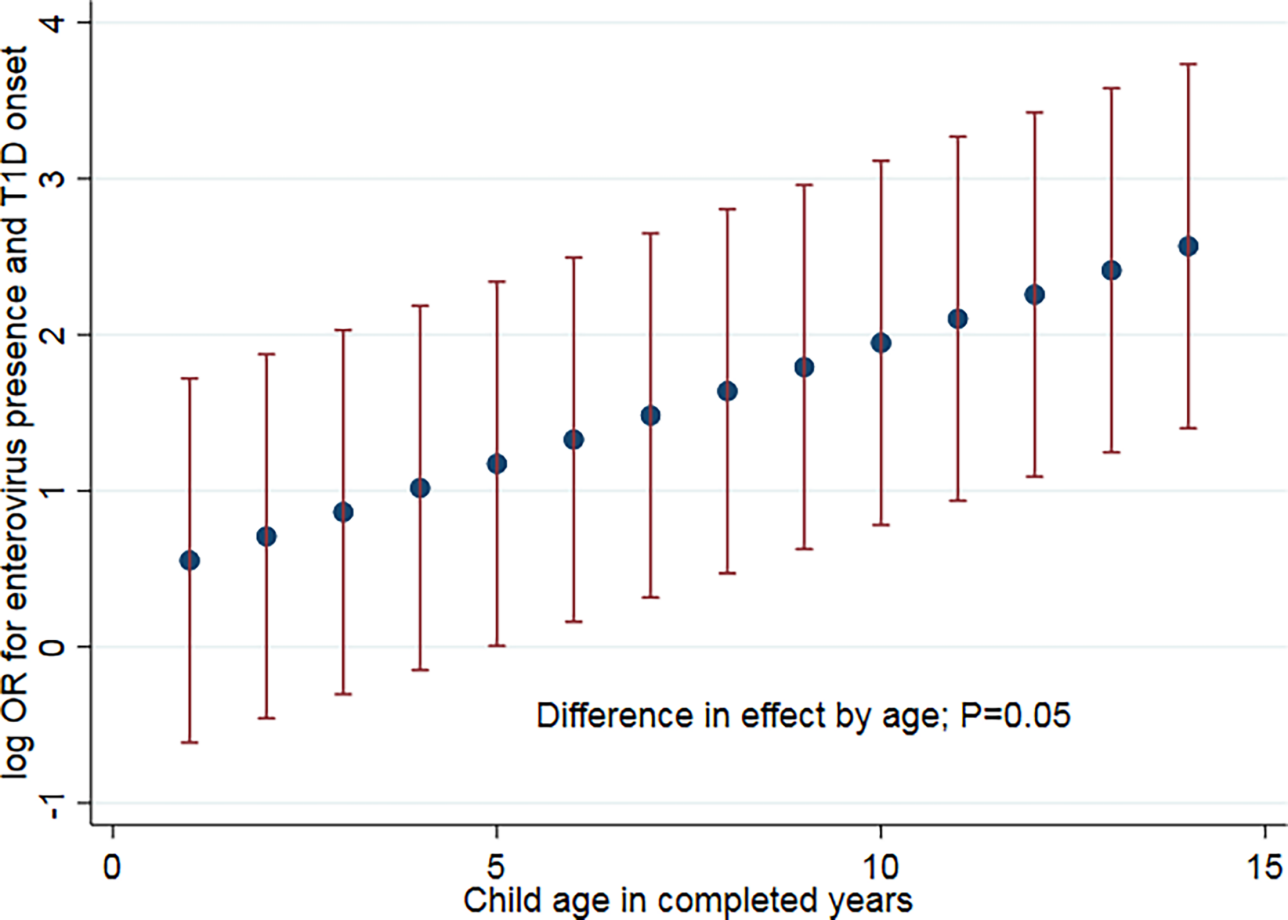
The association between detectable enterovirus and type 1 diabetes onset varies by child age. Linear model: The odds ratio (with 95% confidence interval) for enterovirus presence and T1D onset increased with age (difference in effect, P = 0.03 per year). For infants at 1 year of age the OR was 1.74 (95% CI: 0.54, 5.59); for children aged 5, OR 3.23 (95% CI: 1.01, 10.38); for children aged 12, OR 9.56 (95% CI: 2.98, 30.70).

We examined effect modification by child age. EV infection was associated with a moderate risk of T1D onset for children aged 1–6 years (matched OR 1.48, P *=* 0.002) but a higher risk for children aged 7–15 years (matched OR 6.00, P *=* 0.097); common odds ratio test P *=* 0.98. When examining the linear influence of child age on the magnitude of association between EV presence and T1D onset, for every year beyond age 1, the risk associated with EV presence increased 1.2 fold; P *=* 0.05 (Fig 2).

There was no evidence a non-linear model provided a better fit to the data (P *=* 0.97). There was no difference in effect by child age on the magnitude of association between EV seropositivity, parental occupational social contact, child hand hygiene or day care attendance and T1D onset.

### Sensitivity analyses

We reconducted the analyses for Victorian-born cases with controls weighted to all Victorian live births. The findings were not materially altered. For example, higher parental occupational social contact composite score-T1D onset (composite well score; AOR 0.71 (95% CI: 0.63, 0.80) per category, composite sick score; AOR 0.61 (95% CI: 0.50, 0.73) per category. EV PCR was strongly associated with T1D onset (AOR 5.31 (95% CI: 3.02, 9.35). Again, the mediation analysis demonstrated that a significant proportion of the association between parental occupational well or sick contact was mediated through EV presence and also EV seropositivity, with a lesser magnitude for the later mediation pathway.

## Discussion

Higher parental occupational social contact is strongly associated with a reduction in child T1D risk with consistent dose response trends. The association is mediated partly through a reduction in EV presence in the peripheral blood of the child at T1D onset and, to a lesser extent, associated EV seropositivity. At diagnosis, T1D cases were more likely to have detectable EV in their peripheral blood and elevated EV seropositivity, indicating greater past exposure to EV, than controls.

The observation that this apparent protective association for parental occupational social contact is enhanced by high child hand hygiene before meals is consistent with several scenarios. One notion is that occupational mixing is acting by boosting the parent’s enterovirus immunity and not have gut EV shedding with high hand hygiene than reducing spread by the fecal-oral route [39] within the family. Part of this effect could be mediated by maternal enterovirus antibodies which with can be assumed to be more frequent and at higher titres in mothers with frequent occupational social contacts thus giving better protection against enterovirus infections [8]. It is not consistent with the mechanism of parental occupational social contact acting by the sharing of a beneficial microbiome [40], a protective agent, or shared beneficial immunity [41] In those situations one would expect good child hygiene to be associated with higher T1D risk and to weaken the association between parental occupational social contact and reduced T1D risk but the opposite patterns were actually observed.

It has long been proposed that past conflicting findings on EV and T1D could be explained if age influenced the effect of EV on T1D risk. Population mixing studies on T1D in the UK have indicated that later EV infection was accompanied by more adverse sequelae than early onset infection at a population level [42]. Fig 2 indicates EV infection was more adverse as child age increased. These findings indicate that child age must be taken into account when assessing the role of EV in T1D. Day care attendance was associated with a reduction in T1D risk, despite being a strong determinant of EV infection and seropositivity among controls. The finding that cases or controls attending day care were younger supports the inference that part of the apparent protective association for day care may be due to an earlier age for EV acquisition. The finding that a history of a flu or cold in the past year was positively associated with T1D onset is consistent with a triggering role of infection, as previously proposed [3].

We included a comprehensive set of measures in conjunction with molecular EV indices in a population-based incident T1D case control study. Highly consistent patterns were observed, for example, for nine of the ten occupational contact indices, significant mediation through EV infection was demonstrated. The mediation analysis indicated that the likely temporal pathway was for parental occupational social contact to act before the altered EV indices. Participation rates for both cases and controls were high, over 80%. Various non-causal explanations investigated and excluded, including adjustment for a wide range of confounders. Importantly, parental occupation social contact did not appear to be acting merely by delaying the harvesting the T1D cases, because higher parental occupation contact was not linked to older age of onset. Due to the availability of Victorian perinatal data on almost all live births, we were able to back-weight the sample and found that selection bias due to using a day surgery sampling frame for controls is unlikely to have contributed to these results. False positive findings are unlikely due to the coherence of multiple lines of evidence across the study [43].

The case control study, although it included some prospective perinatal measures, was not fully prospective. However, the window of focus of this investigation was on the time of T1D clinical onset, which would not have been captured for all cases by prospective cohort design with routine follow-up. However, the later study design would have provided an ability to evaluate the role of parental occupational social contact, enterovirus infection and the development of islet autoimmunity, which we could not examine here. Additionally, we were unable to account for genetic influences and measure enterovirus shedding directly; future studies should incorporate these measures where possible. Recall bias is unlikely with regard to the main exposures:- parental occupation and child hand hygiene because current patterns at the time of T1D onset were the focus. However, history of infection over the past year may be more prone to recall bias. The similarity of effect sizes for maternal and paternal effects argue against a strong contribution of in utero effects, which would have required a prospective design. Parental occupational social contact was measured by questionnaire not by a more detailed occupation grid with job duration, yet the non-differential misclassification introduced by this would have tended to move results towards the null but strong associations were observed. The study size is not large, but it was adequately powered to detect the large magnitudes of association evident here and related mediation and interaction. The study did not detect associations between sibling distributions and T1D. This may reflect that, in this setting, only 16% (54/330) of T1D cases were under compulsory school age (6 years) and had not attended day care or other child care outside the home. Thus, sibling-sourced infections may have been overwhelmed by infections sourced from day care, child care, school or parents in this setting.

Previous work on parental occupational social contact and T1D has been limited. The one earlier report found non-significant tendency for mothers with higher occupational social contact to have a reduced risk of T1D onset under 5 years of age [17], consistent with these findings. The occupational social contact patterns found in this study are very similar to those found for herpes zoster prevention [10] where humeral immune boosting against the Varicella-zoster virus in those working with children is thought to be the underlying mechanism. However, this study shows striking transmission across a generation. The findings that cases were more likely to have EV indices in their peripheral blood at diagnosis is consistent with past work, including meta-analysis. Meta-analysis of past studies on day care attendance and T1D have reported significant heterogeneity with a summary odds ratio of 0.6 (95% CI: 0.5, 0.8) for those under 5 years [44]. Our results are consistent with this, probably because children attending day care here were relatively young—80% of T1D cases attending day care were aged less than 5 years.

These findings add to the growing body of evidence that EV presence at T1D onset is important because here, the mediation analysis demonstrated a more distal parental risk factor to be mediated through this more proximal factor. One of the most striking features of T1D onset is that presence of EV by PCR is associated with a summary odds ratio of 9.8 [3]. In the only study to examine fresh pancreatic tissue at diagnosis, EV capsid protein 1 was detected more often (P *=* 0.01) in the islets of 100% (6/6) cases compared to 22% (2/9) of controls, 3–9 weeks after onset [45]. Social network studies confirm two important infectious sources for children are horizontal peer contacts and diagonal adult contacts, with most physical contacts occurring in the home [12]. In the intrafamilial EV study, the higher likelihood of parental EV infection compared to non-family adult controls is noteworthy (OR infinity, P<0.001). In light of the findings here, it may be that the higher EV infection rates among siblings of a T1D case (and subsequent T1D among EV-positive siblings) reflects EV transmission from a parent to multiple children rather than T1D case to sibling transmission. The finding that parental occupational social contact was important as a determinant of case EV indices but not control EV indices again indicates that EV transmission from parents may be particularly adverse compared to EV transmission by other means outside the family such as through day care. Greater hazard associated with parentally-transmitted EV would be consistent with the finding that T1D case mothers having higher EV IgM and IgG antibodies in countries with low T1D incidence rates [43].

In conclusion, higher parental occupation social contact is associated with reduced offspring T1D risk through a reduction in child EV infection. The T1D risk associated with EV presence increased with child age. As good child hand hygiene potentiated the risk reduction associated with high parental occupation contact, these findings are more consistent with protection against parental EV shedding than sharing of a protective infectious agent or microbiome.

## Supporting information

S1 Table Parental occupational social contact questions with mutually exclusive responses. (PDF)
