# Supplementary material for: Higher parental occupational social contact is associated with a reduced risk of incident pediatric type 1 diabetes: Mediation through molecular enteroviral indices
Source: PLoS One. 2018 Apr 17;13(4):e0193992. doi: 10.1371/journal.pone.0193992 (PMC5903611; doi:10.1371/journal.pone.0193992)
Supplement: S1 Table — (PDF) [file pone.0193992.s001.pdf]

S1 Table. Parental occupational social contact questions with mutually exclusive responses

	Mother (or other)	Father (or other)
How many adults do you come into contact with on a usual weekday between 9am-5pm?	☐ No adults<br>☐ <10 well adults<br>☐ 10<30 well adults<br>☐ 30 or more well adults<br><br>and<br>☐ No sick adults<br>☐ 1 or more sick adults	☐ No adults<br>☐ <10 well adults<br>☐ 10<30 well adults<br>☐ 30 or more well adults<br><br>and<br>☐ No sick adults<br>☐ 1 or more sick adults
How many children do you come into contact with on a usual weekday between 9am-5pm?	☐ No children under age 16<br>☐ <10 well children under age 16<br>☐ 10<30 well children under age 16<br>☐ 30 or more well children<br><br>and<br>☐ No sick children<br>☐ 1 or more sick children	☐ No children under age 16<br>☐ <10 well children under age 16<br>☐ 10<30 well children under age 16<br>☐ 30 or more well children<br><br>and<br>☐ No sick children<br>☐ 1 or more sick children
How many animals do you come into contact with on a usual weekday between 9am-5pm?	☐ No animals<br>☐ <10 well animals a day<br>☐ 10<30 well animals a day<br>☐ 30 or more animals a day<br><br>and<br>☐ No sick animals<br>☐ 1 or more sick animals	☐ No animals<br>☐ <10 well animals a day<br>☐ 10<30 well animals a day<br>☐ 30 or more animals a day<br><br>and<br>☐ No sick animals<br>☐ 1 or more sick animals
